# Barrier properties of fungal fruit body skins, pileipelles, contribute to protection against water loss

**DOI:** 10.1038/s41598-021-88148-0

**Published:** 2021-04-22

**Authors:** Klaus J. Lendzian, Andreas Beck

**Affiliations:** 1 Department Pflanzenwissenschaften, Technische Universität München, Wissenschaftszentrum Weihenstephan, 85350 Freising, Germany; 2 Botanische Staatssammlung München, SNSB-BSM, Menzinger Str. 67, 80638 München, Germany; 3 Systematic Botany and Mycology, LMU Munich, Menzinger Str. 67, 80638 München, Germany; 4 GeoBio-Center, LMU Munich, Richard-Wagner-Str. 10, 80333 München, Germany

**Keywords:** Fungi, Biological techniques, Ecology, Plant sciences, Ecology

## Abstract

The permeability of intact fungal fruit body skins (pileipelles) with respect to water and oxygen was determined for the first time. Methods that have been successfully applied to plant surfaces were used to study isolated pileipelles. Mechanically isolated skins from five genera of Basidiomycota (species of *Amanita*, *Russula*, *Stropharia*, *Tapinella*, and *Tricholomopsis*) were mounted between two compartments simulating the inner (fruit body) and the outer (aerial) space. Fluxes of water and oxygen across the skins were measured. Water loss via intact skins differed markedly from evaporation of water from a water surface. The skins reduced water loss by factors of 10 to 30, with permeability ranging from 2.8 to 9.8 × 10^−4^ ms^−1^. Oxygen permeability was much lower and ranged from 0.8 to 6.0 × 10^−6^ ms^−1^. Chloroform-extractable substances play a minor, but significant role as transport barrier during water permeance. Water and oxygen permeability were dependent on the humidity in the aerial compartment. Higher humidity in the air increased permeability and the hydration/water content of the skins. The ecological implications include impacts to fungal growth, sporulation and spore release.

## Introduction

The driving force for the transpirational water loss from living cells is mainly the result of the water potential gradient between cells and the surrounding atmosphere as well as temperature differences^1^. The water potential of the atmosphere decreases rapidly with decreasing humidity and exceeds − 200 MPa at a relative humidity of 20%. This is a hostile environment for cells having a water potential of about − 0.5 to − 5 MPa^2^. It is therefore not surprising that virtually every plant life stage, from germination to vegetative growth and final morphology bears the imprint of adaptations necessary to survive with a limited water supply in the occupied habitat. In adapting to terrestrial environments, aerial parts of plants have evolved water-proofing devices. Higher plants (*Spermatophytina*) have developed the most efficient barriers against water loss and uncontrolled gas exchange, the epidermal cuticle-stomata complex^3–8^ and the analogous periderm-lenticel complex^9,10^. The existence of a cuticle or cuticle-like structures in terrestrial lower plants, such as the *Bryophyta* (mosses), is still under debate^11^ but at least lipophilic compounds seem to provide a superficial protective layer of mostly unknown efficiency^12^.

At first glance the aerial parts of fungal fruit bodies, especially the Basidiomycota/Agaricomycotina (the mushrooms), have not evolved special structural features for the fungi-atmosphere interfaces to cope with the varying water potential differences. Cuticle-like structures or cell layers similar to an epidermis of higher plants are not formed. However, fungal fruit bodies (called basidiomes) may show skin surface structures like parallel irregular hyphae or a strongly geliferous pileipellis which have been interpreted as contributing to barrier properties^13^. The mycelium of the fruit body is generally multicellular and compacted into a pseudoparenchymatous tissue, the plectenchyma which is covered by a multicellular pileipellis. The fruit body emerges into the air and is associated with the differentiation of reproductive structures and the dissemination of spores^14–16^. In general, the water content of the fruit bodies is high, and their appearance in natural habitats is frequently correlated with the presence of moisture^17^. Both, high humidity and moisture content of the substrate are important for the formation of fruiting bodies^18^. Dry matter is usually in the range of 6–14% of fresh weight^19^. Obviously, most fleshy mushrooms are adapted to humid conditions, but small deviations in air moisture from complete saturation lead to differences in water potentials which could increase water loss from the fruit bodies, highlighting the importance of any possible water retaining structures. At relative humidity (RH) of 96% and 90% and 20 °C, the water potential of the atmosphere reaches values of − 5.5 MPa and − 14.2 MPa, respectively^1^. Such negative values are supposed to induce and enhance transpiration of the aerial parts of the mushrooms as their mean water potentials are reported to vary from − 1.5 to − 2.5 MPa^20^. Consequently, water-proofing devices could lower the water loss significantly and have an important impact on the biology of the mushroom.

To date, transpiration rates of mushrooms have been measured under various conditions using several methods. However, the exchange of oxygen and carbon dioxide, important physiological gases, has not been examined. Water loss has been recorded either by weighing the isolated fruit body^21,22^ or by registering the amount of water diffused into the atmosphere^23,24^. Attempts to quantify water permeability led to conflicting results which were the result of experimental difficulties and inadequacies. The driving force for transpirational water loss has not been determined or defined explicitly. One problem is that the area of the gills is difficult to determine exactly. In contrast, the outer layers of the caps, exposed directly to the atmosphere, can often be removed as a peel but has received no attention with regard to its transport properties for gases like oxygen and carbon dioxide or water vapour. The outer layers of the cap are exposed to the turbulent part of the atmosphere where differences in the water potential of fruit bodies and air are highest.

Therefore, we tested the hypothesis that the pileipellis constitutes a significant water-barrier, decreasing transpirational water loss of the fungal fruit bodies. At the same time, oxygen permeance is reduced as well. To this end, a new approach was used to quantify the permeability of pileipelles with respect to water and oxygen. Well adapted methods and insights from the studies with plant cuticles and periderm were applied. The aim was to assess the importance of structural, biophysical and chemical features such as fungal lipids and water content for the survival of fruit bodies in hostile environments.

## Materials and methods

### Fungal material

Fungal fruit bodies of *Amanita muscaria* (L.) Lam.*, Russula cyanoxantha* (Schaeff.) Fr.*, Stropharia aeruginosa* (Curtis) Quél.*, Tapinella atrotomentosa* (Batsch) Šutara*,* and *Tricholomopsis rutilans* (Schaeff.) Singer were collected directly from natural habitats, i.e. from the forests west of the city of Freising (Bavaria, Germany). Collection took place early in the morning. Common species were selected in order to harvest enough material for experiments. Time did not allow criteria such as age and developmental stage to be taken into consideration, but are left for a later study. Each fruit body was cut off with a sharp knife and was transported to the laboratory in boxes at a relative humidity always higher than 98%. Care was taken to collect only specimens with minimal to no dust or particle contamination. Squares with a side of 2.0 cm were cut from the central area of the upper surface of the caps and stripped off carefully with forceps. These isolated parts will be termed fungal skin (FS) or pileipellis. The fungal fruit body skins were isolated about 1 h after harvest. Only skins which could be stripped off easily and without damages were accepted as intact and suitable for experiments. They were further checked for imperfections like cracks or holes under the stereoscope; imperfect ones were discarded. Without further treatment the isolated skins were immediately used for the experiments. Each pileipellis was subjected to the following successive procedures: determination of water and oxygen permeance, extraction of lipids with chloroform, second determination of water and oxygen permeance.

### Determination of water permeance P_W_

Water permeance P_W_ (m s^−1^) is defined as the quotient of water flux (mass per area and time; mg m^−2^ s^−1^) and driving force, which is the difference in the chemical potential of water between the inner and the outer surface of the fruit body skin. For simplicity we defined the driving force as water vapour saturation concentration of 23.05 g m^−3^ at 25 °C^10,25,26^. That is the maximum quantity of water a dry atmosphere (RH = 0%) can take up from a liquid water reservoir during equilibration. This absorbed water quantity is negatively correlated to RH. All permeances given in this communication are vapour based.

Isolated pileipelles were mounted on water-filled (0.6 ml) transpiration chambers made of brass as described^27^. The morphological inner side of the skins were faced towards the bulk water (donor). Water could only leave the chambers via the skins and water loss was followed gravimetrically with a microbalance (accuracy ± 0.1 mg, Sartorius Göttingen, Germany) for 2 h. The area of the single pileipellis exposed to water was 0.785 cm^2^. Chambers were kept upside down bringing the skin into contact with the bulk water. Between weighing events (every 10 min for 10 s) the transpiration chambers were kept at 25 °C in closed compartments (receivers) containing sufficient dry silica gel to maintain the relative humidity and water activity within the compartments constant at 3% and 0.03, respectively. To adjust the water activity within these compartments to a defined value, dry silica gel was replaced by salt solutions. Saturated salt solutions supplied relative humidities between 93% (sodium carbonate) and 19% (lithium chloride) corresponding to water activities of 0.93 and 0.19, respectively^28^. Air pressure was recorded but did not influence the data, as shown by preliminary experiments. Uncovered transpiration chambers, filled with water, were exposed directly to the aerial compartment and served as controls for water loss.

After the determination of water and oxygen permeance, the skins were carefully removed from the transpiration chambers and were air dried. Then they were dipped into an excess of chloroform for 5 min to extract chloroform soluble material^29^. In the context of this study, the chloroform soluble compounds are termed fungal lipids throughout, and have not been further analysed. Effects on physiological properties of the pileipelles have not been investigated yet. After extraction the chloroform was allowed to evaporate from the skins (30 min). The chloroform-extracted skins, designated as EFS (extracted fungal skin), were remounted on the transpiration chambers and permeances were determined again over silica gel.

### Determination of oxygen permeance P_O_

The permeability of fruit-body skins for oxygen was determined according to the method described for plant cuticles^4^. Briefly, the isolated skins were mounted between a gaseous and an aqueous compartment with the morphological outer side (aerial side) of the skin facing the gaseous compartment. In a second set of experiments the aqueous compartment was replaced by a gaseous one. During the experiments, one compartment saturated with oxygen (100% oxygen gas or oxygen saturated water) and designated as the donor was separated by the pileipellis from the receiver containing an oxygen electrode (Orbisphere Labs, Geneva, Switzerland) which continuously recorded the amount of oxygen permeating the fungal membrane. The water activity within the receiver compartment was kept constant either by silica gel or by salt solutions. The mounted compartments were kept in a water bath at 25 °C. Permeance P_O_ was calculated from mass oxygen flux and the difference in oxygen concentration across the single skin^30^. If possible, the same intact samples were used to determine both oxygen and water permeance.

To determine oxygen permeance before and after extraction of chloroform-soluble material samples were handled as described above.

### Sorption of water vapour

Sorption of a molecule by a barrier is the first step during the permeation process^1^. The sorption of water molecules from humid air by pileipellis samples was followed gravimetrically using a closed, evacuable ultra-microbalance (accuracy ± 100 ng; model S3D-V, Sartorius Göttingen, Germany). Pileipelles were dried over silica gel to a constant mass and were kept in the desiccator until experimentation. The electronic balance and the registration devices were located in a temperature- controlled room at 25 °C^31^. Each measurement was preceded by a calibration cycle. Immediately after this procedure, the system containing the balance was opened. The dry skin, trimmed to a 1-mg-piece, was mounted onto the platin hook which replaced the scale pan, and the system was closed again. The dry mass of the sample was registered. These operations usually took less than 2 min. The measurement of the sorption was started by allowing the defined humid air to enter the balance chamber within a few seconds. Humid air with a defined water content at 25 °C was supplied continuously from salt solution/air mixtures^28^. The increase in mass of the skin, due to sorption of water, was followed until a constant reading was reached. The same piece of skin was used to measure water sorption for the entire humidity range, starting with the lowest. The experiments were repeated using 34 samples of each mushroom species.

### Statistics

Permeances P_W_ and P_O_ for non-extracted and chloroform-extracted skins were always determined on the same membrane and could be paired up for comparison (paired random samples). As the test statistic followed a normal distribution the paired sample t-test was applied using SPSS (SPSS Inc., Chicago, USA). Sample sizes for each species varied between 23 and 38 (exact sample sizes are given in Figures and Tables). Replications of an experiment on the same pileipellis sample were impossible because the extraction with chloroform changed the original barrier properties. Consequently, the number of individual samples used was as high as possible. Arithmetic means with 95% and 99% confidence intervals were calculated and incorporated into the tables.

### Consent to participate

Both authors consent to participate.

### Consent for publication

Both authors publish by mutual consent.

## Results

In order to quantify the water permeability of the upper fruit body cap skin, an in vitro system was selected using the isolated skin (pileipellis). Preliminary experiments with 28 samples for each mushroom species resulted in permeances for water that varied by more than one order of magnitude within the limits of 4 × 10^−3^ ms^−1^ and 1 × 10^−4^ ms^−1^. The highest permeances coincided with the use of transpiration chambers lacking a covering skin. The lowest permeances clearly indicated that there were barrier properties in isolated fungal skins. Several reasons may lead to the wide range of permeances: a) skins intrinsically have a broad variability in losing water; b) removal of the skins partly results in undetectable defects; c) very small holes caused by insects and other predators lead to another but effective pathway for water loss. These problems with in vitro systems have been discussed previously for isolated plant cuticles and their transport parameters^26^.

**Figure 1:**
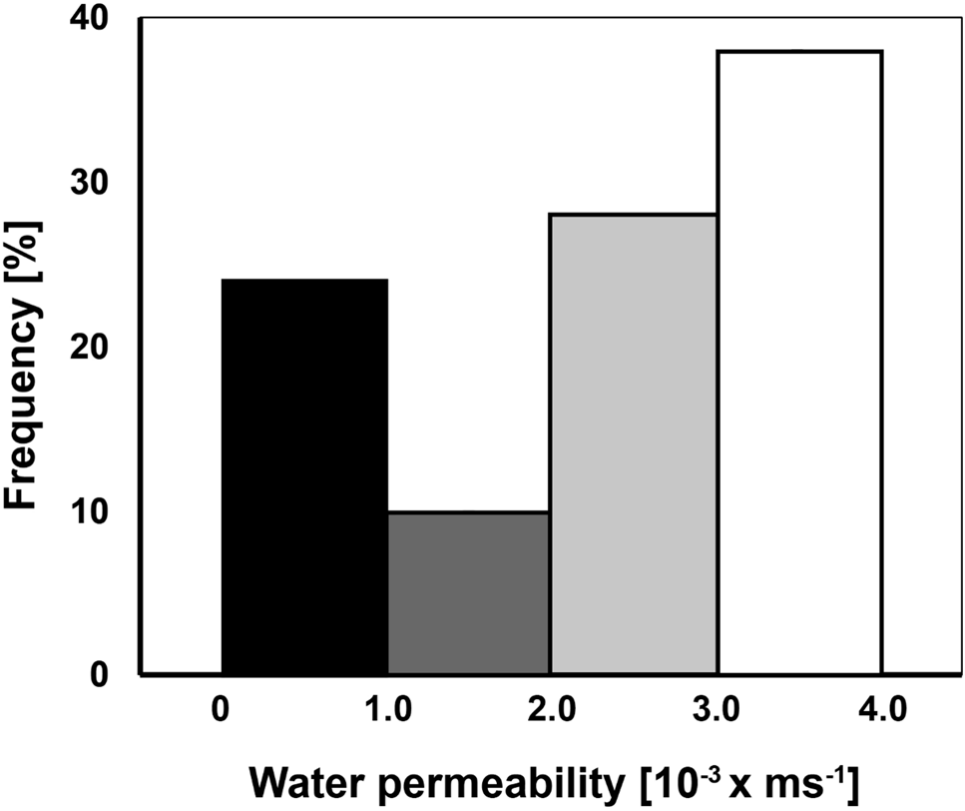
Frequency of vapour phase-based water permeances P_W_ of all fungal cap skins (peleipelles) isolated from the five collected species (n = 140; 28 from each species). Four clusters represent the following ranges for P_W_ (ms^−1^ × 10^3^): 4–3; below 3–2; below 2–1; below 1–0.1. The amount of all skins measured has been set to 100%

Water permeances did not differ significantly among species, and ranged between 0.1 and 4 × 10^−3^ ms^− 1^. Plotting values of permeances of all species against their frequency revealed a distribution with a broad variation (Fig. 1). This is also known for cuticles of higher plants^32,33^. The majority (38%) of the determined P_W_ values from all mushroom species clustered in a range from 4 to 3 × 10^−3^ ms^−1^. Water loss and the corresponding P_W_ values from uncovered transpiration chambers fell into the same range, indicating that the skins were either heavily damaged during isolation or had undetected holes. 28% and 10% of the P_W_ values fell into the ranges below 3 to 2 × 10^−3^ ms^−1^ and below 2 to 1 × 10^−3^ ms^−1^, respectively. Thus, these skins showed some barrier characteristics which became pronounced in range below 1 to 0.1 × 10^−3^ ms^−1^ where 24% of the values were found. Skins with P_W_ values below 1 × 10^−4^ ms^−1^ have never been observed. Clustering P_O_ values for oxygen resulted in a similar distribution of abundance showing lowest P_O_ within the range 7 × 10^−6^ ms^−1^ and 0.7 × 10^−6^ ms^−1^ (data not shown). Permeances lower than 0.7 × 10^−6^ ms^−1^ have not been detected. In both cases pileipelles with the lowest permeability were clearly distinguishable from the other ones. Skins from this category were judged intact, and were used in further experiments.

Water loss via intact fruit body skins differed markedly from evaporation of water from an uncovered water surface at comparable driving forces (Table 1). While the permeance of a water surface for water amounted to 3.4 × 10^−3^ ms^−1^ P_W_ values of the intact skins lay within the range of 0.28 × 10^−3^ ms^−1^ and 0.98 × 10^−3^ ms^−1^. Mean values for P_W_ differed among the investigated genera by a maximum factor of 3.5. Compared to the values observed on isolated plant cuticles^33^, coefficients of variation between different skins of a particular mushroom species were noticeable small, pointing to a high reproducibility of the measured values. The barrier against water loss could be partly removed by extracting the skins with chloroform which resulted in significantly increased permances (Table 1). In all cases, the remaining structures showed measurable resistances to the transfer of water. The chloroform-extractable material, termed fungal lipids, made up 1.5% (w/w) of the dry skins, but it has not been further analysed chemically.

**Table 1:** Vapour phase-based water permeances P_W_ (ms^−1^ × 10^4^).

Species	sample size	Permeance		EFS/FS
		FS	EFS	
*Stropharia aeruginosa*	34	2.8 (21%)	9.1 (13%)	3.3**
*Tapinella atrotomentosa*	32	3.6 (11%)	9.8 (22%)	2.7**
*Russula cyanoxantha*	32	6.3 (24%)	21 (11%)	3.3**
*Amanita muscaria*	34	7.5 (31%)	11 (27%)	1.5*
*Tricholomopsis rutilans*	34	9.8 (19%)	19 (16%)	1.9**

P_W_ values are given for dry skins from selected fungal caps before (FS) and after (EFS) extraction with chloroform. P_W_ values of the extracted skins differ significantly at the significance level α ≤ 5% (*) or α ≤ 1% (**). Confidence intervals for permeances are given in parentheses.

Isolated pileipelles were also permeable to oxygen (Table 2) but permeances were significantly lower. The average P_O_ values for oxygen were about two orders of magnitude below those measured for water. The isolated skins became more permeable upon removal of the chloroform-soluble fraction (Table 2). However, with one exception these changes were not large and statistically not significant.

**Table 2:** Vapour phase-based oxygen permeances P_O_ (ms^−1^ × 10^6^).

Species	sample size	Permeance		EFS / FS
		FS	EFS	
*Tapinella atrotomentosa*	32	0.8 (17%)	1.1 (13%)	1.4**
*Stropharia aeruginosa*	34	2.3 (29%)	2.8 (16%)	1.2
*Russula cyanoxantha*	32	2.7 (30%)	3.5 (15%)	1.3
*Amanita muscaria*	34	3.4 (31%)	4.4 (26%)	1.3
*Tricholomopsis rutilans*	34	6.0 (13%)	6.6 (11%)	1.1

P_O_ values are given for dry skins from selected fungal caps before (FS) and after (EFS) extraction with chloroform. P_O_ values of the extracted skins differ significantly at α ≤ 1% (**). Confidence intervals for P_O_ are given in parentheses.

Both, water and oxygen permeance were determined by applying the largest concentration difference (largest driving force) across the skins, i.e. water or 100% oxygen gas at the donor side and dry air or dry nitrogen gas, respectively, at the receiver side. According to the equation:

$${\text{Mass Transfer F }}\left( {{\text{mg m}}^{{ - {2}}} {\text{s}}^{{ - {1}}} } \right) \, = {\text{ Permeance }}\left( {{\text{m s}}^{{ - {1}}} } \right) \, \times {\text{ Driving Force }}\Delta {\text{c }}\left( {{\text{mg m}}^{{ - {3}}} } \right)$$

it is assumed that permeance is constant and F can be calculated for any given driving force. This held true for P_O_ values determined for oxygen under the experimental conditions mentioned. At any given concentration gradient of oxygen across the fruit body skin, the permance P_O_ resulted in the same value, this being constant for a single species (data not shown). For example, oxygen transfer across the skin from *Tapinella atrotomentosa* yielded a value of 1.14 mg O_2_ m^−2^ s^−1^ or 36 μmol O_2_ m^−2^ s^−1^ at the maximum concentration gradient (100% O_2_, equivalent to 1428 g m^−3^). From an atmosphere containing 21% oxygen about 0.24 mg O_2_ m^−2^ s^−1^ can be transferred via the skin, assuming a theoretic oxygen level near zero within the fruit body. Corresponding values can be calculated for different oxygen gradients and different mushroom genera.

**Figure 2:**
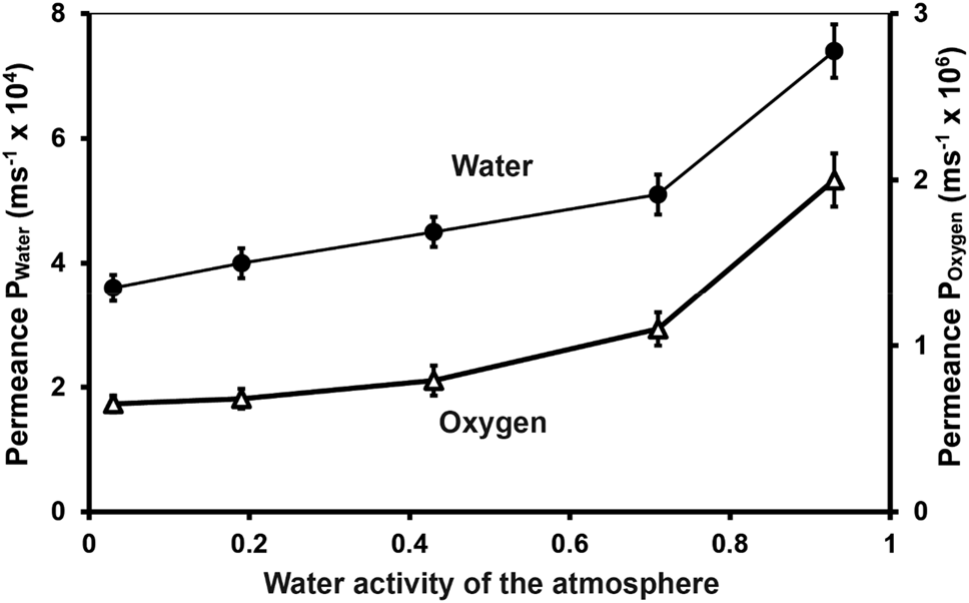
The influence of changing water activities in the atmosphere (in the receiver compartment of the measuring device) on the vapour phase-based oxygen and water permeance of 38 isolated cap skins of *Tapinella atrotomentosa.*

In contrast, P_W_ values for the transfer of water across the skins was not constant. Changes in the water potential gradient introduced by increasing the water activity from near zero (dry air) to almost water vapour saturated air (high humidity) resulted in significantly higher P_W_ values (Fig. 2). Those changes were especially pronounced in the range of higher humidities. Over the whole range P_W_ changed by a factor of two and in a typical way. This is illustrated for *Tapinella atrotomentosa* in Fig. 2, but also holds true for the other genera investigated, resulting in analogous curves.

Variable P_W_ values for the water transfer implies that the barrier characteristics of the skins can be affected by the water potential within the pileipellis. The changing characteristics could be indirectly observed when oxygen permeance was determined at increasing humidity in the receiver compartment, i.e. at the aerial side of the skin (Fig. 2). P_O_ was shown to be independent of the oxygen concentration gradient across the skins, but was dependent on the presence of water. Increasing humidity at the surface of the aerial side of the skin resulted in an elevation of P_O_ by a factor of two. The dependency along the humidity scale was almost identical to that of P_W_. The most pronounced increase of permeance was observed with humidities above 70%. In both sets of experiments, the water activity was increased from 0.03 to 0.93. Lowering water activity again from 0.93 to 0.03 led to similar results; thus, a hysteresis could be excluded.

**Figure 3:**
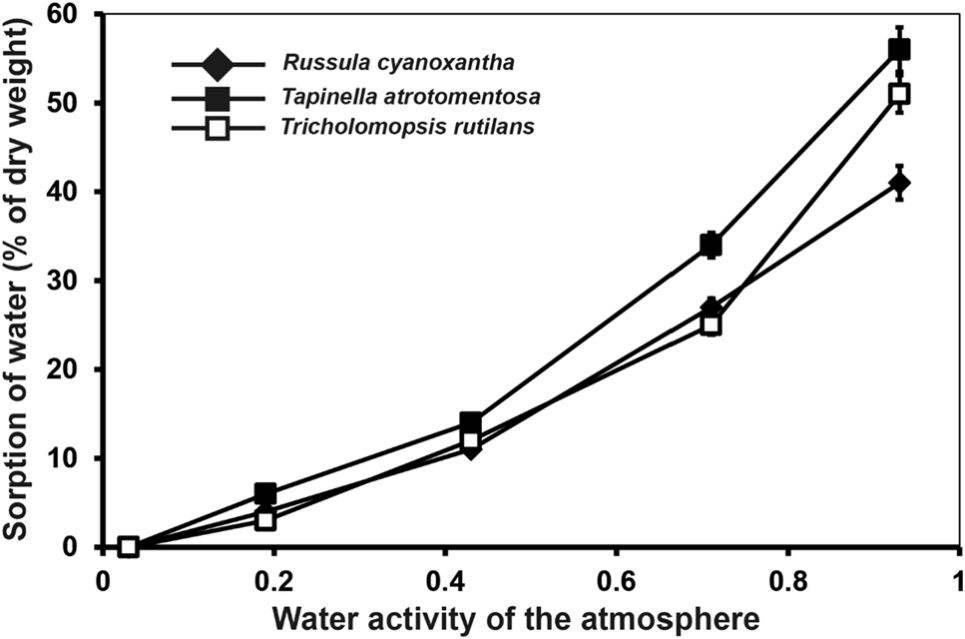
Sorption of water vapour by isolated, dehydrated cap skins (23 skins of each species) from an atmosphere with increasing water activities. The amount of water sorbed after equilibration is given as % increase in weight; the initial weight of the dehydrated skins was always trimmed to 1 mg.

Isolated dry fruit body skins took up water from a humid atmosphere (Fig. 3, shown for representatives of three genera only). The amount of water sorbed plotted against humidity followed an exponential equation and did not differ significantly among all genera investigated. Sorbed water amounted up to 40–60% (w/w) of the skins at a humidity of 93% and at equilibrium. All data represent values determined after equilibrium had been reached. Sorption of water vapor was completely reversible and showed no significant hysteresis upon decreasing the humidity of the atmosphere. Time courses for the sorption and desorption processes are not included in this paper. But in general, each equilibrium was established within 2 h.

The water content of the skins had a remarkable impact on the permeances for water and oxygen (Fig. 3). To further demonstrate the importance of this observation, water loss via a defined skin area and within a defined time period at different humidities of the atmosphere, was calculated (Table 3). Assuming P_W_ is constant it becomes obvious that decreasing humidities and increasing water deficits of the atmosphere result in increasing losses of water from the fungal fruit body. However, with variable P_W_ values, correlated to variable air humidities, water loss becomes significantly reduced.

**Table 3:** A comparison of variable permeances P_W_ to a constant one with respect to water loss at varying relative humidity RH. Data are taken from Fig. 2 and represent those of the species *Tapinella atrotomentosa* . Calculation of water loss F is based on the equation F = P_W_ × Δc.

RH	Δc	P_W_	Loss of water F (mg m^−2^ s^−1^)	
(%)	(mg m^−3^)	(m s^−1^)	P_W_ = variable	P_W_ = 7.4 × 10^−4^
93	1614	7.4 × 10^−4^	1.19	1.19
71	6685	5.1 × 10^−4^	3.41	4.94
43	13,139	4.5 × 10^−4^	5.91	9.72
19	18,671	4.0 × 10^−4^	7.47	13.82
3	23,050	3.6 × 10^−4^	8.30	17.06

## Discussion

As with all aspects of environmental stress, organisms can either avoid or tolerate the adversity. Avoidance of desiccation stress can be achieved either by buffering mechanisms at the morphological or physiological level or else by timed development, so that sensitive tissues are not exposed at the period of greatest desiccation risk. Tolerance of drought stress implies that tissues are actually dehydrated to an acceptable degree only. Fruit bodies of the Basidiomycota seem to emerge into the aerial space only when soil and atmosphere contain moisture near at saturation^18,34^. While the soil water content changes only slowly over time, the humidity of air can decrease rapidly within a few hours after a rain event or during rising temperatures. Generally, such changes occur frequently within the life time of fruit bodies and driving forces for the loss of water become larger than those retaining water in the organisms^15,22,35,36^.

The numeric data for the permeances of water and oxygen presented in this pilot study clearly demonstrate that the skins of the upper part of the fruit bodies of selected mushrooms represent resistances, which remarkably reduce their loss from the bodies. If the skins are compared with cuticles^26^ and phellem^10^ of higher plants, then the skins provide only weak barriers to water and oxygen permeability. In general, the differences in permeances between mushrooms and higher plants amount to at least three to four orders of magnitude. Nevertheless, water loss via the pileipellis is about 10 to 30 times lower (based on unit area and driving force) than that of an uncovered water surface.

The permeance P_W_ represents the relationship between flux (F) of water (per area and time) and the concentration gradient as driving force for the flux (P_W_ = F/Δc). In pure physical systems permeance P_W_ is constant, when only Δc and nothing else changes. That holds true for P_O_ and the fungal skins. On the other hand, P_W_ is not a constant and depends on the gradient of the water concentration (water activity) across the skins (Fig. 2, Table 3). In situ*,* one has to assume that the fruit body is saturated with water when young and vital. This water is used for keeping hymenia moist which is crucial for spore liberation. Barrier characteristics of the fungal skin come into play with changing relative humidity in the air, and results in a lower water loss under dry conditions than would be expected at a constant P_W_ (Table 3). Due to the variability of P_W_ water loss via the fruit body skins at relative humidities below 50% (water activity below 0.5) is reduced by a factor of around 2.

**Figure 4:**
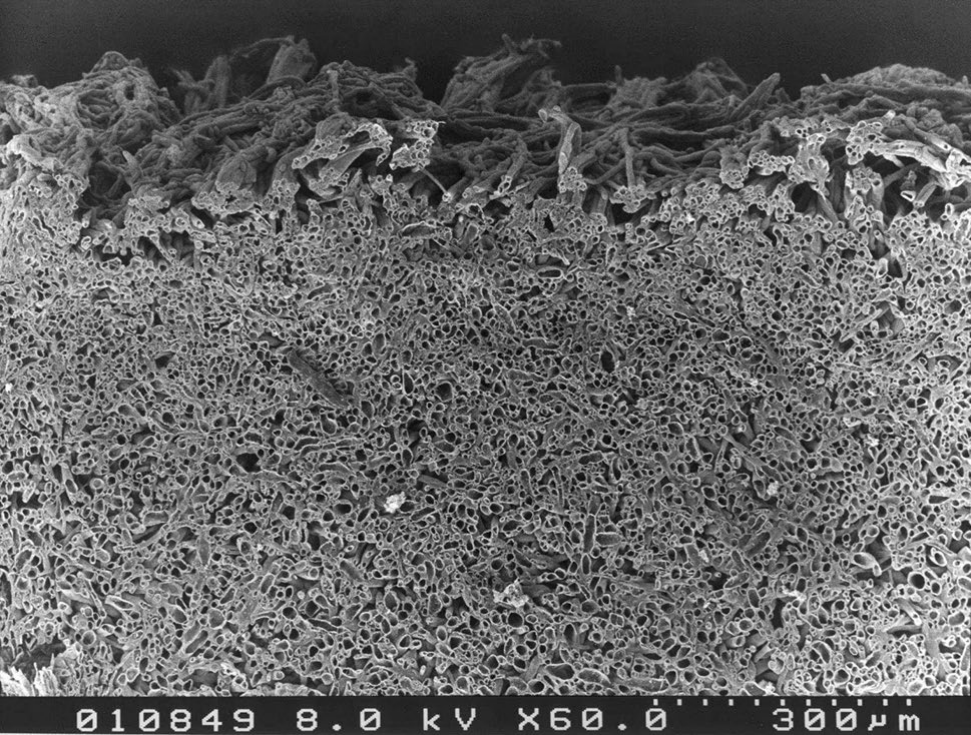
Scanning electron micrograph of a cross section of *Tapinella atrotomentosa* pileipellis. The outer surface/aerial side of the pileipellis is facing to the top. The specimen was prepared as reported^46^.

It can be assumed that in situ*,* the first phase of water loss is the diffusion of water from the plectenchyma cells into the intercellular space. This transfer is hindered by the cell walls and probably by the lipids. Another hindrance for water vapour diffusion from the fruit body into the free air space is represented by the very narrow intercellular spaces in the pileipellis. From the data in Fig. 4 space diameters can be estimated to be less than 10 µm on average. Occasionally, diameters reach up to 30 µm. The relationship between diameter and length of the pathway to the surface, substantiates the idea that the intercellular space represents a narrow and long diffusion pathway and is thus a diffusion barrier^1^. Long and narrow diffusion pathways are also recognized as being involved in the transfer of water via the cuticles of higher plants^26^. Given the water diffusion resistance of the pileipellis, transpirational water loss is directed to the gills, where evaporation is essential to enable ballistic spore release^37,38^. This increased condensation of water onto the hygroscopic hilar appendage at the base of the spores, is necessary for efficient spore discharge^39^.

The ecological significance of fruit body skins in mushrooms is obvious. During rain events and high humidity in the air, mushrooms have no problems with respect to their water balance. Under these conditions the barriers within the skins of the fruit bodies are sufficient for survival (e.g. spore production). At lower humidities in the atmosphere, the fruit bodies lose increasing amounts of water but due to the variable permeance of the skins, the water loss becomes relatively reduced. Some water loss, however, is beneficial for mushrooms, as it has been shown that evaporation cools the surface^40^. Temperature differences in the direct vicinity of the fruit body^41^ lead to increased airflows below the cap and support efficient spore dispersal.

The barrier properties of the investigated skins are directly correlated to their water content. Completely dry skins sorb increasing amounts of water with increasing water activities in the environment (Fig. 3). The actual water content of the skins directly influences the permeance of water and oxygen (Fig. 2). Water is not only a permeant for the fungal skins but serves directly as a modulator for the diffusion of water and oxygen within the skins. In many biological systems the uptake of water results in hydration and swelling of vacuoles, protoplasts, and cell walls. These processes are reversible^1^. It is supposed that similar processes occur within the fungal skins. With increasing water contents, the hyphae and their cell walls swell to form a normal plectenchyma with intercellular spaces. Upon dehydration turgor decreases and cell walls shrink, resulting in a kind of structural collapse which is not fatal but reversible. In this situation, the intercellular space may become reduced resulting in a reduced water flux from the interior of the fruit body into the aerial environment. This may result in a longer diffusion path for water under drying conditions, as it needs to be recruited from more inner parts of the pileipellis in order to evaporate. But under wet conditions evaporation is increased, which is beneficial as it has been also shown that it is important to prevent the hymenium from getting soaked^15,42^.

None of the skins investigated showed more negative permeances than those given in Table 1 and Table 2. The measured fluxes of water and oxygen point to a remarkable barrier, and the stripped skins seem to present the in vivo barrier conditions of the upper cap surface. However, that observation does not necessarily mean that these permeances truly reflect the in vivo barrier conditions. The stripped fungal skins under investigation had a mean and uniform thickness of about 600 µm. Scanning electron micrographs show a dense and compact network of hyphal cells (plectenchyma; Fig. 4). The intercellular space is reduced to a minimum. At the surface, the hyphal system loses its compactness and disperses, resulting in a much fissured surface. In order to calculate permeances, one has to carefully determine the surface area. Figure 4 demonstrates, that this is almost impossible and indicates, that the barrier properties are underestimated when just a water surface is used for comparison. Further investigations on the structure of the isolated skins have to be conducted. Hydrophobins are well known to be important in fruiting body formation^16,43^, and lipids seem to be part of the transport resistances for water and oxygen. Their removal by chloroform resulted in a significant increase in permeance for water, and less pronounced for the oxygen transport. That observation confirms the importance of lipids in the biological activity of the pileipelles. The chemical nature of the lipids has not been investigated yet, but chloroform extracts very apolar components. Extraction with more polar solvents like methanol and ethanol did not change permeances. These results give no information about the localization of the lipids within the skins. The simplest model assumes that they cover the surfaces of the internal hyphae adjacent to the intercellular space. Such protective interfaces between living organisms and the aqueous or gaseous environment have been established since the beginning of life on earth^44,45^. In all cases the interfaces are of lipid nature.

Since the water potential of the atmosphere is mostly much lower than that of living organisms, fungi, plants and animals have had to evolve and cope with this situation. To protect against constant water loss, all living organisms developed quite similar barrier strategies, and the Basidiomycota seem to be no exception.
